# Validation of Plasma p-tau217 as a Biomarker of Prodromal Alzheimer’s Disease

**DOI:** 10.31083/RN50619

**Published:** 2026-07-21

**Authors:** Victoria Monge-García, Sofía Lorenzo-García, María-Carmen Bernal-Soriano, Patricia Torrella-Esteban, Sonia Monge-García, José Sánchez-Payá, José-Antonio Monge-Argilés

**Affiliations:** ^1^Physical Medicine and Rehabilitation Section, Marina Baixa Hospital, 03570 La Vila Joiosa, Alicante, Spain; ^2^Sanitary and Biomedical Research Institute (ISABIAL), 03010 Alicante, Spain; ^3^Department of Clinical Analysis, University General Hospital Dr. Balmis, 03010 Alicante, Spain; ^4^Department of Neurology, St. Augustinos Hospital, 52355 Düren, Germany; ^5^Preventive Section, University General Hospital Dr. Balmis, 03010 Alicante, Spain; ^6^Department of Neurology, University General Hospital Dr. Balmis, 03010 Alicante, Spain

**Keywords:** validation, plasma biomarker, plasma p-tau 217 protein, prodromal Alzheimer’s disease, CSF, reproducibility

## Abstract

**Background:**

Plasma phosphorylated tau 217 protein (p-tau217p) has recently been proposed as a useful biomarker for the early diagnosis of Alzheimer’s disease (AD). However, local validation is recommended because of the potential influence of clinical, analytical, and preanalytical factors on assay performance.

**Methods:**

Between 2021 and 2024, we evaluated patients with amnestic mild cognitive impairment (aMCI) through clinical history, neurological and neuropsychological examination, blood sampling for biobanking, brain imaging, and lumbar puncture, among other diagnostic tests. In September 2025, p-tau217p levels were measured simultaneously using the LUMIPULSE immunoassay (Fujirebio). The diagnostic validity, reproducibility, receiver operating characteristic (ROC) curve performance, correlation with cerebrospinal fluid (CSF) biomarkers, and influence of clinical and analytical variables were evaluated in this study.

**Results:**

Among the 108 aMCI patients included, 66 met the criteria for clinic-biological AD, while the remainder had alternative clinical diagnoses. Using a two-threshold approach, p-tau217p levels ≥0.19 yielded a sensitivity of 88% and a positive predictive value of 83% for identifying AD. Levels ≥0.39 showed a specificity of 91% and a positive predictive value of 90% for the same purpose. Intermediate values (0.20–0.38) achieved a specificity of 83%. The intraclass correlation coefficient for the assay reproducibility was 0.97. The ROC curve for p-tau217p demonstrated an area under the curve of 0.86 for diagnosing AD. P-tau217p correlated more strongly with CSF p-tau181 (ρ = 0.63; *p* < 0.01) than with CSF Aβ1-42 (ρ = –0.40; *p* < 0.01). Finally, a reduced glomerular filtration rate was associated with a significant increase in p-tau217p levels (*p* < 0.006).

**Conclusions:**

In our setting, p-tau217p measurement showed a high validity for the diagnosis of prodromal AD, which is consistent with the recent neurological literature. The assay showed high reproducibility, although results may be influenced by renal function. P-tau217p correlated more strongly with CSF p-tau181 than with CSF Aβ1-42.

## 1. Introduction

The diagnostic framework for Alzheimer’s disease (AD) has undergone a profound transformation in recent decades, evolving from a purely clinical construct [1] to the currently accepted clinic-biological model [2]. Historically, the biological dimension relied on abnormalities detected through brain positron emission tomography (PET) and cerebrospinal fluid (CSF) biomarkers [3]. The hallmarks alterations in CSF include reduced Aβ1-42 concentrations (or a decreased Aβ1-42/Aβ40 ratio) and elevated p-tau181 levels [4].

Recently, robust evidence has demonstrated that plasma p-tau isoforms—particularly p-tau217 (p-tau217p)—exhibit strong diagnostic validity for AD, including during the prodromal phase [5,6,7]. This growing body of data has led to the incorporation of p-tau217p into the latest diagnostic criteria proposed by the Alzheimer’s Association [8], raising the possibility that invasive and costly procedures, such as CSF analysis and PET imaging may become unnecessary in a substantial proportion of patients [8].

However, the literature reveals two partially divergent lines of investigation regarding the p-tau217p. Some studies have focused on its ability to detect cerebral amyloidosis [9,10], whereas others have evaluated its accuracy in identifying AD pathology itself [11,12]. These objectives are not interchangeable, as amyloid deposition may also occur in other clinical entities, such as dementia with Lewy bodies [13,14,15,16], even at prodromal stages [13,17].

Another point of debate concerns the biological interpretation of p-tau217p. While some authors consider it primarily a marker of amyloid-related-processes [9,10], others interpret it as a more direct indicator of tau pathology [5,18]. This conceptual ambiguity, together with the variability observed in clinical practice, has prompted the development of guidance documents to support the appropriate use and interpretation of p-tau217p [19].

Given these uncertainties and the need for local accurate diagnostic performance, the current neurological literature recommends establishing local analytical capacity for p-tau217p quantification, as several analytical and pre-analytical factors remain critical for ensuring reliable implementation [20,21,22]. Accordingly, our primary objective was to determine the diagnostic validity of this technique within our clinical setting.

Multiple analytical platforms have been evaluated [5,18], and among automated systems, Lumipulse has emerged as one of the most widely adopted and diagnostically robust [5,11,12,18].

### Objectives

The primary objective of this study was to determine the diagnostic accuracy of p-tau217p for AD in a cohort of 108 patients with amnestic mild cognitive impairment (aMCI) who underwent CSF biomarker assessment in our department, with plasma and CSF samples obtained simultaneously for analysis. The CSF biomarker criteria served as the reference standard. To address the secondary objectives, we repeated the assay in 25 samples to evaluate the reliability of the technique and examined the correlation between p-tau217p and CSF Aβ1-42 and p-tau181, which are considered specific biomarkers of amyloidosis and tauopathy, respectively.

## 2. Materials and Methods

### 2.1 Study Design

This was a retrospective observational cohort study (see **Supplementary Material**).

### 2.2 Study Population

We included patients diagnosed with aMCI who attended the Dementia Clinic of Hospital General Universitario Dr. Balmis. All individuals underwent a standardized diagnostic work-up, including medical history, general and neurological examination, neuropsychological assessment, blood testing, brain imaging (mostly magnetic resonance imaging (MRI)), and lumbar puncture (LP) between 2021 and February 2024. At the time of LP, a blood sample was obtained for plasma separation and measurement of the p-tau217p level.

### 2.3 Inclusion Criteria

• Age >55 years.

• Mini-Mental State Examination (MMSE) score ≥24.

• Signed Biobank informed consent for CSF and blood collection.

• Barthel Index ≥95.

• Lawton–Brody Instrumental Activities of Daily Living score ≥4.

### 2.4 Exclusion Criteria

• Established dementia.

• Neurological, psychiatric, or medical disorders that could account for cognitive impairment.

• Absence of Biobank informed consent.

• Anticoagulated patients.

• Depression with a Yesavage Geriatric Depression Scale score >5.

• Score >2 on the Fazekas MRI criteria.

### 2.5 CSF Collection

All CSF samples were collected between 10:00 and 14:00. An experienced neurologist performed the LP using a 20 × 3.5 gauge needle. CSF was collected in standard polypropylene tubes, centrifuged for 10 min at 1500 g, and subsequently aliquoted into polypropylene tubes. The samples were stored at –80 °C. Only samples containing fewer than 50 red blood cells were included in the analysis.

### 2.6 Measurement of Core AD Biomarkers in CSF

Core AD biomarkers (Aβ1-42, total tau, and p-tau181) were quantified using a commercial ELISA kit (P23354736, Innotest, Innogenetics/Fujirebio, Ghent, Belgium) following the manufacturer’s instructions. Analyses were performed blinded to the clinical diagnosis within six months of LP.

The normal reference values were defined as follows:

• Aβ1-42 >800 pg/mL.

• Total tau <350 pg/mL.

• p-tau181 <56.5 pg/mL.

Abnormal Aβ1-42 and p-tau181 values are required to classify a CSF profile as consistent with AD, according to the 2018 NIA-AA criteria [3].

### 2.7 Measurement of p-tau217p

Blood samples were collected in EDTA-K2 tubes (SARSTEDT 23, Numbrecht, Sajonia, Germany) and subsequently centrifuged (2000 rpm × 10 min, 4 ºC) within 2 hours after extraction. Plasma was aliquoted and stored at –80 ºC until analysis. In September 2025, all plasma samples were measured simultaneously on the Lumipulse fully-automated platform G600II, using commercially available kits for p-tau217p (81472, Fujirebio Europe, Ghent, Belgium). On the day of analysis, the plasma samples were brought to room temperature, mixed thoroughly, centrifuged for 5 min at 2000 ×g, and subsequently transferred to specific cuvettes for analysis on the Lumipulse platform (version 12, Fujirebio, Ghent, Belgium). A few days later, the assay was repeated with 25 samples to assess the reproducibility of the test.

Lumipulse G pTau 217 Plasma is an immunoassay for the quantitative measurement of threonine-217 phosphorylated tau (p-tau217p) in K2-EDTA plasma samples. The assay is based on chemiluminescent enzyme immunoassay (CLEIA) technology using a specific two-step method on the LUMIPULSE G system (version 3, Fujirebio, Ghent, Belgium). Its two-step design provides high sensitivity and specificity by employing two antibodies (capture and detection) that bind to the same antigen, thereby markedly reducing false-positive results.

### 2.8 Statistical Analysis

To describe the characteristics of the patients included in the study, quantitative variables—given their non-parametric distribution verified with the Kolmogorov–Smirnov test—were summarized using the median and the 25th and 75th percentiles. Qualitative variables were summarized as absolute frequencies and relative percentages for each category. Comparisons of quantitative variables between groups (Alzheimer’s disease vs. non-Alzheimer’s) were performed using the Mann–Whitney U test, whereas associations with qualitative variables were examined using the chi-square test or, when required, Fisher’s exact test.

For the reliability analysis, the Intraclass Correlation Coefficient was calculated, and a Bland–Altman plot was generated. To assess the diagnostic validity of p-tau217p levels for Alzheimer’s disease, the area under the receiver operating characteristic curve (AUC-ROC) and its 95% confidence intervals (95% CI) were computed. Subsequently, for different p-tau217p cut-off points, the following metrics were estimated with their corresponding 95% CIs: Sensitivity, Specificity, Positive Predictive Value (PPV), Negative Predictive Value (NPV), Positive Likelihood Ratio, Negative Likelihood Ratio, and Youden’s Index.

To examine the association between p-tau217p levels and CSF Aβ1-42 and p-tau181 concentrations, Spearman’s correlation coefficient was used. Group comparisons of p-tau217p levels were conducted using the Mann–Whitney U test when two groups were evaluated (e.g., history of myocardial infarction) or the Kruskal–Wallis test when three groups were compared (e.g., apolipoprotein E (*APOE) *genotype). Variables showing statistically significant associations in the bivariate analyses were subsequently entered into a multiple linear regression model to adjust for potential confounders. All analyses were performed using IBM SPSS Statistics version 25 (IBM Corp., Armonk, NY, USA), and statistical significance for all hypothesis tests was set at *p* < 0.05.

### 2.9 Study Limitations

The main limitation of this study was its single-center design. Nonetheless, this characteristic will allow us to compare our findings with existing literature to determine whether this technique can be reliably implemented in our population. Otherwise, we have a relatively limited sample size and we need a broader and prospective cohort to definitively validate our results.

## 3. Results

Table 1 summarizes the comparison of demographic, clinical, and analytical characteristics between the two study groups. Patients with AD were slightly older (*p* < 0.05), had a higher proportion of women (*p* < 0.001), and showed a greater frequency of *APOE* ε4 genotypes (*p* < 0.001). In contrast, non-AD patients exhibited higher MMSE (*p* = 0.002) and higher Neuropsychiatric Inventory (NPI) (*p* < 0.006) scores. Finally, AD patients showed lower CSF Aβ1-42 levels (*p* < 0.0001) and higher CSF p-tau181 (*p* < 0.0001) and p-tau217p levels (*p* < 0.0001) compared with non-AD individuals. No significant differences were observed in the other clinical or analytical variables.

**Table 1.** **Demographic, clinical, and laboratory characteristics of the included patients**.

		AD (n = 66)	No-AD (n = 42)	*p*
Age; median (p25–p75)		75 (72–77)	73 (67–76)	0.048
Gender (male); n (%)		18 (27.3)	28 (66.7)	<0.001
Glomerular filtration (mL/min); n (%)				0.169
	≥90	15 (22.7)	16 (38.1)	
	89–60	37 (56.1)	21 (50.0)	
	≤59	14 (21.2)	5 (11.9)	
History of myocardial infarction; n (%)		3 (4.5)	4 (9.5)	0.151
History macroscopic brain lesion; n (%)		8 (12.1)	2 (4.8)	0.198
High blood pressure n (%)		37 (56.1)	21 (50.0)	0.538
Hypercholesterolemia n (%)		31 (47.0)	24 (57.1)	0.303
Diabetes mellitus n (%)		11 (16.7)	9 (21.4)	0.535
*APOE* genotype n (%)				<0.001
	ε2	0 (0.0)	3 (7.1)	
	ε3	29 (43.9)	35 (83.3)	
	ε4	37 (56.1)	4 (9.5)	
MMSE; median (p25–p75)		25 (24–27)	27 (25–28)	0.002
Categorical verbal fluency; median (p25–p75)		12 (10–15)	13 (11–15)	0.343
Barthel scale; n (%)				0.476
	100	50 (75.8)	30 (71.4)	
	95	10 (15.2)	5 (11.9)	
	≤90	6 (9.1)	7 (16.7)	
Lawton-Brody scale; median (p25–p75)		6 (5–8)	5 (4–8)	0.158
Neuropsychiatric inventory; median (p25–p75)		4 (2–6)	6 (4–8)	0.006
CSF Aβ1-42 protein (pg/mL); median (p25–p75)		666 (554–806)	1053 (731–1295)	<0.001
CSF p-tau181 protein (pg/mL); median (p25–p75)		104 (79–136)	37 (25–46)	<0.001
p-tau217 protein (pg/mL); median (p25–p75)		0.43 (0.27–0.63)	0.12 (0.09–0.21)	<0.001

AD, Alzheimer’s disease; No-AD, no Alzheimer’s disease; MMSE, Mini Mental State Examination; *p*, significance level; p25–p75, percentile 25 and 75; CSF, cerebrospinal fluid; *APOE*, apolipoprotein E.

**Fig. 1.**
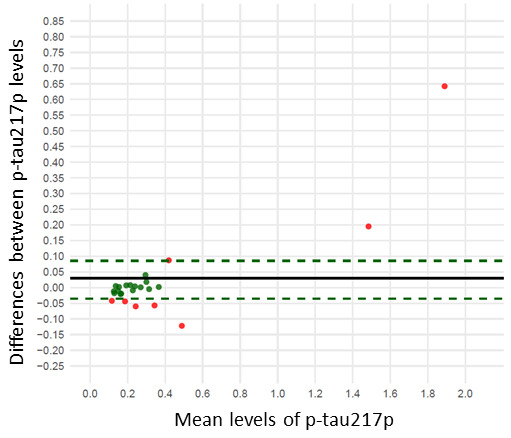
**Reproducibility of p-tau217p in 25 AD patients**. Green dots indicate samples with differences between repeated measurements within the 95% limits of agreement, reflecting good reproducibility. Red dots represent outliers with differences exceeding the 95% limits of agreement.

In Fig. 1, we show the reproducibility of p-tau217p in 25 AD patients. Intraclass Correlation Coefficient (ICC) was 0.97 (95% CI: 0.93–0.98).

**Fig. 2.**
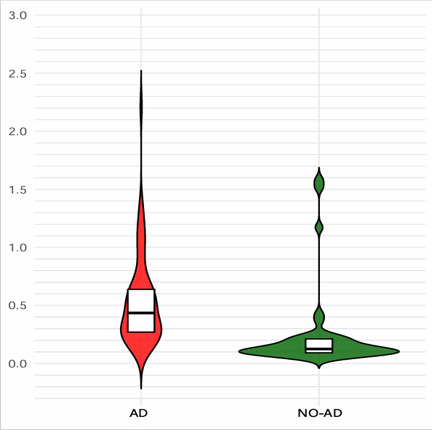
**p-tau217p protein levels in both groups are included**.

Fig. 2 illustrates the distribution of p-tau217p concentrations in the two study groups, demonstrating a statistically significant difference (*p* < 0.001).

**Fig. 3.**
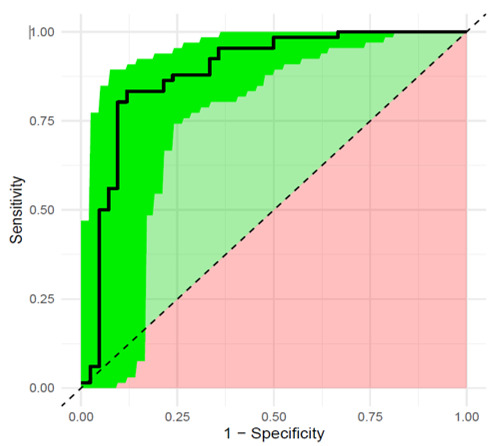
**Receiver operating characteristic (ROC) curve illustrating the diagnostic performance of p-tau217p for prodromal AD**.

Fig. 3 shows the diagnostic accuracy of p-tau217p for AD, yielding an AUC of 0.86 (95% CI: 0.78–0.95).

Table 2A presents the diagnostic performance of p-tau217p using the four local predefined cut-off points. Sensitivity exceeded 80% for thresholds below 0.24 pg/mL, whereas specificity surpassed 80% for values at or above 0.24 pg/mL. The positive predictive values (PPV) were consistently ≥80%, while the negative predictive values (NPV) did not reach 80% for any cut-off. 28.7% of the cases fell into the intermediate zone.

**Table 2A.** **Diagnostic performance of p-tau217p across multiple concentration thresholds. Local four cut-offs**.

p-tau217p levels (pg/mL)		≥0.160	≥0.212	≥0.242	≥0.420
Sensitivity; % (95% CI)		89.4 (81.2–97.6)	84.8 (75.4–94.2)	83.3 (73.6–93.0)	53.0 (40.2–65.8)
	(TP/TP+FN)	(59/59+7)	(56/56+10)	(55/55+11)	(35/35+31)
Specificity; % (95% CI)		64.3 (48.6–80.0)	76.2 (62.1–90.2)	85.7 (73.9–97.5)	90.5 (80.4–100.0)
	(TN/TN+FP)	(27/27+15)	(32/32+10)	(36/36+6)	(38/38+4)
PPV; % (95% CI)		79.7 (69.9–89.6)	84.8 (75.4–94.2)	90.1 (81.8–98.5)	89.7 (78.9–100.0)
	(TP/TP+FP)	(59/59+15)	(56/56+10)	(55/55+6)	(35/35+4)
NPV; % (95% CI)		79.4 (64.4–94.5)	76.2 (62.1–90.2)	76.6 (63.4–89.7)	55.0 (42.6–67.5)
	(TN/TN+FN)	(27/27+7)	(32/32+10)	(36/36+11)	(38/38+31)
Youden index		0.5 (0.4–0.7)	0.6 (0.4–0.7)	0.7 (0.5–0.8)	0.4 (0.3–0.6)
Positive likelihood ratio		2.5 (1.7–3.8)	3.5 (2.0–6.1)	5.8 (2.7–12.3)	5.6 (2.1–14.5)
Negative likelihood ratio		0.2 (0.1–0.3)	0.2 (0.1–0.3)	0.2 (0.1–0.3)	0.5 (0.4–0.7)

95% CI, percentage and 95% confidence interval; TP, true positive; FN, false negative; FP, false positive; TN, true negative; PPV, positive predictive value; NPV, negative predictive value.

Table 2B shows the diagnostic validity of p-tau217p using the two-threshold approach. Levels ≥0.19 pg/mL yielded sensitivity and PPV values above 80%, whereas levels ≥0.39 pg/mL achieved specificity and PPV values of ≥90%. For intermediate values (0.20–0.38 pg/mL), only the specificity exceeded 80% and 25% of the cases fell into this intermediate zone.

**Table 2B.** **Diagnostic performance of p-tau217p across the two-threshold approach**.

p-tau217p levels (pg/mL)		≥0.199	0.200–0.389	≥0.39
Sensitivity; % (95% CI)		87.8 (79.2–96.5)	30.3 (18.4–42.1)	56.0 (43.3–68.8)
	(TP/TP+FN)	(58/58+8)	(20/20+46)	(37/37+29)
Specificity; % (95% CI)		71.4 (56.6–86.2)	83.3 (70.8–95.7)	90.5 (80.4–100)
	(TN/TN+FP)	(30/30+12)	(35/35+7)	(38/38+4)
PPV; % (95% CI)		82.8 (73.3–92.4)	74.0 (55.6–92.4)	90.2 (79.9–100)
	(TP/TP+FP)	(58/58+12)	(20/20+7)	(37/37+4)
NPV; % (95% CI)		78.9 (64.6–93.2)	43.2 (31.8–54.6)	56.7 (44.1–69.3)
	(TN/TN+FN)	(30/30+8)	(35/35+46)	(38/38+29)
Youden index		0.6 (0.4–0.7)	0.14 (–0.02–0.29)	0.47 (0.3–0.6)
Positive likelihood ratio		3.0 (1.9–5.0)	1.82 (0.84–3.92)	5.9 (2.2–15.3)
Negative likelihood ratio		0.2 (0.1–0.3)	0.84 (0.68–1.03)	0.49 (0.3–0.6)

Table 3 summarizes the influence of clinical and analytical variables on p-tau217p concentrations in both groups. A lower glomerular filtration rate was significantly associated with higher p-tau217p levels. Likewise, the presence of at least one *APOE* ε4 allele was associated with increased p-tau217p concentrations compared with the other genotypes. None of the remaining variables reached statistical significance, for that reason, they were not included in the adjusted model.

**Table 3.** **Influence of clinical and laboratory variables on p-tau217p concentrations**.

		Median (p25–p75)	*p*	*p*a
Glomerular filtration (mL/min)			0.006	0.019
	≥90	0.14 (0.09–0.46)		
	89–60	0.28 (0.15–0.52)		
	≤59	0.36 (0.24–0.93)		
*APOE* genotype			0.049	0.039
	ε2	0.08 (0.07–n.c.)		
	ε3	0.20 (0.11–0.49)		
	ε4	0.36 (0.26–0.64)		
History of myocardial infarction			0.830	-
	Yes	0.19 (0.11–0.85)		
	No	0.28 (0.14–0.54)		
History of macroscopic brain injury			0.221	-
	Yes	0.57 (0.20–0.66)		
	No	0.26 (0.13–0.51)		
High blood pressure			0.536	-
	Yes	0.27 (0.14–0.61)		
	No	0.27 (0.12–0.52)		
Diabetes mellitus			0.133	-
	Yes	0.18 (0.12–0.42)		
	No	0.30 (0.14–0.56)		
Hyperlipidemia			0.487	-
	Yes	0.24 (0.14–0.48)		
	No	0.31 (0.13–0.62)		

n.c., not calculable.

**Fig. 4.**
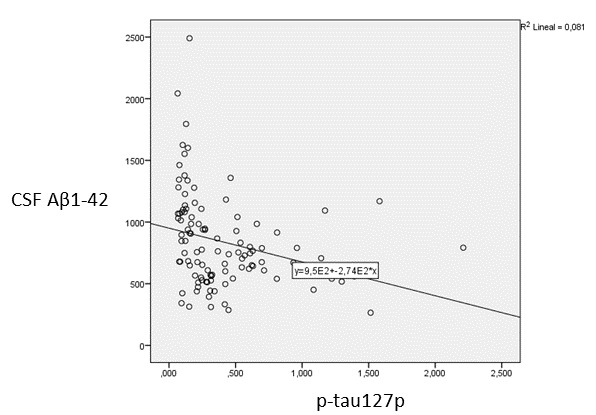
**Linear correlation between CSF Aβ1-42 protein concentrations and the p-**t**au217p levels**. Each dot represents an individual patient. The regression line indicates a negative association, albeit modest (R^2^ = 0.081), demonstrating a negative correlation with Aβ1-42 (ρ = –0.40; *p* < 0.01).

**Fig. 5.**
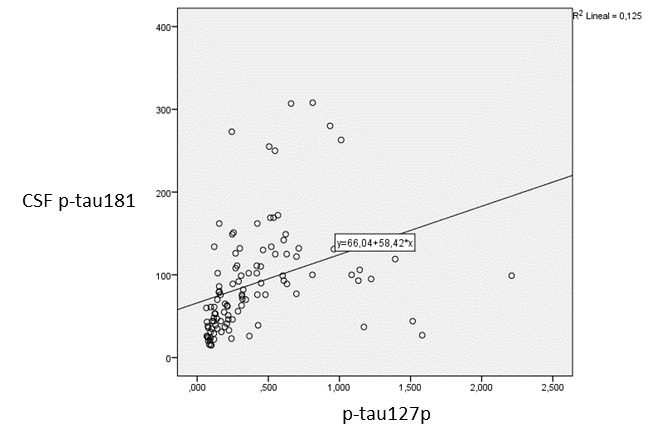
**Linear correlation between CSF p-tau181 concentrations and the p-tau217p levels**. Each dot represents an individual patient. The regression line (y = 66.04 + 58.42 x) indicates a positive association, albeit modest (R^2^ = 0.125), suggesting that higher p-Tau217p levels are weakly associated with increased relative tau phosphorylation.

Fig. 4 shows the correlation between p-tau217p and CSF biomarkers, demonstrating a negative correlation with Aβ1-42 (ρ = –0.40; *p* < 0.01) and a positive correlation with p-tau181 (ρ = 0.63; *p* < 0.01) (Fig. 5).

Finally, in Table 4, we show the statistical differences between AD and no-AD patients, taking into account the final clinical diagnosis. Given that the subjective memory complaints subgroup is the most numerous within the non-AD cohort, we highlight its differences in comparison with the AD group (see **Supplementary Table 1**).

**Table 4.** **Comparison of p-tau217p levels between AD and no-AD patients**.

	Median (p25–p75)	*p* (respect to AD levels)
Alzheimer’s disease (n = 66)	0.43 (0.27–0.63)	-
Lewy body dementia (n = 3)	0.18 (0.10–n.c.)	0.014
Subjective memory impairment (n = 21)	0.14 (0.09–0.21)	<0.001
Psychiatric illness (n = 4)	0.12 (0.09–1.16)	0.129
Frontal dementia (n = 9)	0.11 (0.07–0.19)	<0.001
Vascular dementia (n = 5)	0.11 (0.10–0.24)	0.001

## 4. Discussion

The diagnostic performance of p-tau217p in our cohort for identifying prodromal AD closely aligns with previously published evidence [5,6,7,11,12]. Current clinical practice guidelines [19] define acceptable triage accuracy as ≥90% sensitivity and ≥75% specificity, whereas confirmatory testing requires ≥90% for both metrics when used as a reference standard in the diagnostic evaluation of suspected AD. In our setting, applying the two-threshold strategy [11,12] yielded sensitivity values approaching 90% for concentrations ≥0.19 pg/mL and a specificity of 91% for concentrations ≥0.39 pg/mL, supporting the feasibility of using this assay as a supportive or triage biomarker within a multimodal or sequential diagnostic framework.

Using locally derived cutoffs, sensitivity similarly approached 90% for p-tau217p levels >0.16 pg/mL, while specificity reached 91% for values >0.42 pg/mL. We recommend the two-cutoff approach, as overall accuracy increased by 4.7% due to cases falling within the intermediate zone, proportions consistent with prior reports [12].

These findings underscore the potential of p-tau217p as a diagnostic biomarker — either as a stand-alone or as part of a sequential two-step workflow alongside PET or CSF biomarkers, offering a cost-effective, scalable, and minimally burdensome strategy for identifying AD. Incorporating p-tau217p into tailored screening pathways may also enhance participant-selection efficiency even for preclinical AD trials [23].

Glomerular filtration rate and *APOE* genotype were significant in both univariate and adjusted analyses. Despite limitations related to sample size and the unicentric design, we consider that both variables should be taken into account when interpreting p-tau217p results, as reduced glomerular function and the presence of at least one *APOE* ε4 allele are associated with higher levels of this protein.

The comparison between the two study groups revealed significant differences in age, sex, *APOE* genotype, and cognitive status. The age difference was minimal and is unlikely to be clinically meaningful. Regarding sex, core CSF biomarkers of Alzheimer’s disease are not known to differ by sex [3], making plasma differences similarly improbable. As expected, the *APOE* ε4 genotype was more prevalent in the AD group, and cognitive status was poorer in AD, given that most non-AD participants were ultimately diagnosed with subjective memory complaints.

The non-AD group is highly heterogeneous, limiting the interpretability of comparisons between the AD group and each individual non-AD subgroup. Nevertheless, significant differences emerged in all subgroup comparisons except for the psychiatric illness group. This pattern may suggest the presence of preclinical AD in at least some individuals within this subgroup.

Confirming the presence of AD pathology [5,6,7,11,12,19] appears to provide greater diagnostic validity than confirming amyloid positivity alone [9,10,24], particularly given that prodromal dementia with Lewy bodies may exhibit amyloid co-pathology even at early stages [13,17]. In our cohort, only one of three Lewy body dementia cases showed p-tau217p levels of 0.21 pg/mL, while the remaining two were below 0.16 pg/mL. These intermediate values, within the two-threshold framework, reinforce the need for confirmatory testing in selected cases, including those presenting with an amnestic phenotype [12].

The reproducibility of the p-tau217p Lumipulse assay was exceptionally high, representing the first report to document this level of analytical stability. Comparable reproducibility has been demonstrated for other platforms, including ALZpath p-tau217 [5], the Eli Lilly assay [25], and the Pittsburgh plasma p-tau217 assay [26]. Additional evidence of robustness comes from studies showing high precision and diagnostic accuracy of p-tau217 across fully automated and semi-automated platforms [27].

Although modest, the stronger correlation between p-tau217p and CSF p-tau181 compared with CSF Aβ1-42 suggests that p-tau217p may function more as a biomarker of tauopathy—or at least as a mixed-pathology marker—contrary to authors who classify it exclusively as an amyloid marker [9,10,24].

The use of p-tau217p in specialized clinical settings is increasingly recommended due to its potential to reduce reliance on more invasive and costly diagnostic procedures [22,28]. Its implementation in primary care has also been proposed [19], although consensus remains limited because of the heterogeneous requirements for its deployment [29]. In such settings, diagnostic validity would differ, particularly regarding PPV and NPV, given the variation in disease prevalence across clinical environments. Moreover, p-tau217p has been significantly associated with cognitive and functional decline in AD, though further studies are needed to validate these associations across diverse populations and to clarify its utility for early-stage detection and longitudinal monitoring [29].

Finally, recent evidence indicates that p-tau217p measurement from dried blood spots may offer a minimally invasive alternative for biomarker assessment [30]. If confirmed in independent cohorts, these findings would provide additional support for the robustness and scalability of this biomarker.

## 5. Conclusions

In conclusion, p-tau217p showed a high validity for diagnosing prodromal AD in our clinical setting, consistent with prior literature. The assay demonstrated excellent reproducibility, although concentrations may be influenced by renal function and *APOE* genotype. The stronger correlation with CSF p-tau181 than with CSF Aβ1-42 may further support its role as a biomarker more closely aligned with tau pathology.

## Data Availability

The datasets used and analyzed during the current study are available from the corresponding author on reasonable request.

## Abbreviations

p-tau 217p, Plasma p-tau217 protein; aMCI, Amnestic mild cognitive impairment; AD, Alzheimer’s disease; CSF, Cerebrospinal fluid.
